# Spinning Into Surgery: A Case of Penile Fracture Due to High-Risk Sexual Activity

**DOI:** 10.7759/cureus.94158

**Published:** 2025-10-08

**Authors:** Hector R Gonzalez-Carranza

**Affiliations:** 1 Urology, Hospital Angeles Metropolitano, Mexico City, MEX

**Keywords:** penile fracture, penile trauma, porn consumption, sexual intercourse, vigorous sexual activity

## Abstract

Penile fracture is a rare but significant urological emergency characterized by rupture of the tunica albuginea of the erect penis, which commonly occurs during vigorous sexual activity. While typical causes vary geographically, sexual intercourse and forceful penile manipulation are the leading etiologies in Western and Eastern countries, respectively. Pornography consumption has been associated with engagement in high-risk sexual behaviors and positions, potentially increasing the risk of injury.

A 54-year-old man presented with acute penile pain, detumescence, and swelling following sexual intercourse in an unconventional position inspired by online pornography, colloquially referred to as the “helicopter” position. Physical examination revealed significant ecchymosis and a palpable defect on the ventral side of the penile shaft. Penile duplex ultrasound confirmed a fracture of the tunica albuginea in the proximal third of the penis, associated with an 8.5 mL hematoma and apparent involvement of a branch of the dorsal penile artery. The patient underwent prompt surgical repair, including hematoma evacuation and primary closure of the tunica defect. At the two-year follow-up, the patient was contacted and evaluated, with no evidence of erectile dysfunction or postoperative complications. This case highlights the role of pornography in promoting risky sexual behaviors, which may contribute to penile trauma in men. Surgical management remains the gold standard for penile fractures, offering favorable functional and cosmetic outcomes when performed promptly. Awareness of the potential risks of unconventional sexual positions and the impact of problematic pornography use is crucial for prevention. Penile fractures require immediate clinical attention to prevent long-term complications. This case underscores the need for public education on safe sexual practices, particularly in the context of increasing exposure to risky behaviors through pornography.

## Introduction

Penile fracture is a urological emergency caused by the fracture of the tunica albuginea of the erect penis, usually during vigorous sexual activity. This condition requires prompt diagnosis and intervention to prevent long-term complications, such as erectile dysfunction or penile deformity [1,2]. The etiology varies worldwide; sexual intercourse and forceful manipulation or the taqaandan maneuver are the most common causes in Western and Eastern countries, respectively [3].

Sexual trauma is commonly associated with the position, being the most frequent “doggy style position” (DSP) and “man on top” position. Clinically, patients typically report a sudden cracking sound, immediate pain, rapid detumescence, and penile swelling or hematoma, often resulting in the characteristic “eggplant deformity,” also known as the aubergine sign [4].

Pornography use has been linked to an increased risk of penile fracture due to exposure to high-risk sexual behaviors and vigorous activity. The accessibility of pornography and the normalization of extreme or acrobatic sexual positions can influence individual sexual practices, often reducing the perception of physical risk [5]. Although the DSP remains the most frequently reported position in cases of penile fracture, less conventional positions, such as the one involved in this case, should not be overlooked as potential contributors to injury.

## Case presentation

**Figure 1:**
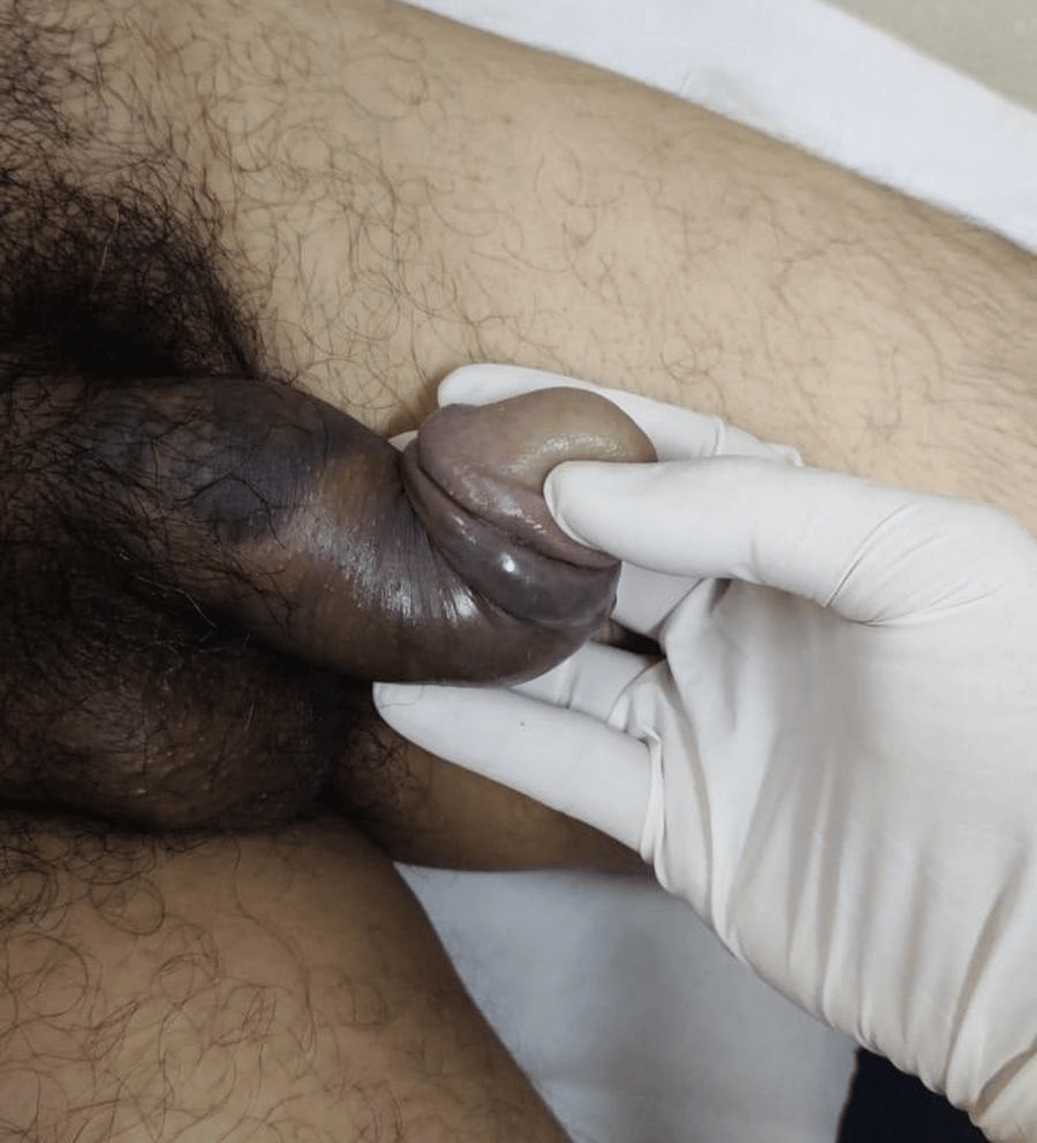
Penile fracture presenting with classic "eggplant deformity" and ecchymosis.

We present a case of a 54-year-old male who arrived at the emergency department with acute penile pain and swelling immediately following sexual intercourse. The patient reported pain as 8/10 on a visual analog scale. He reported hearing a cracking sound during sexual intercourse, followed by immediate detumescence and rapid swelling. He had tried a new sexual position after seeing a pornographic film called "the helicopter" (Figure 1).

**Figure 2:**
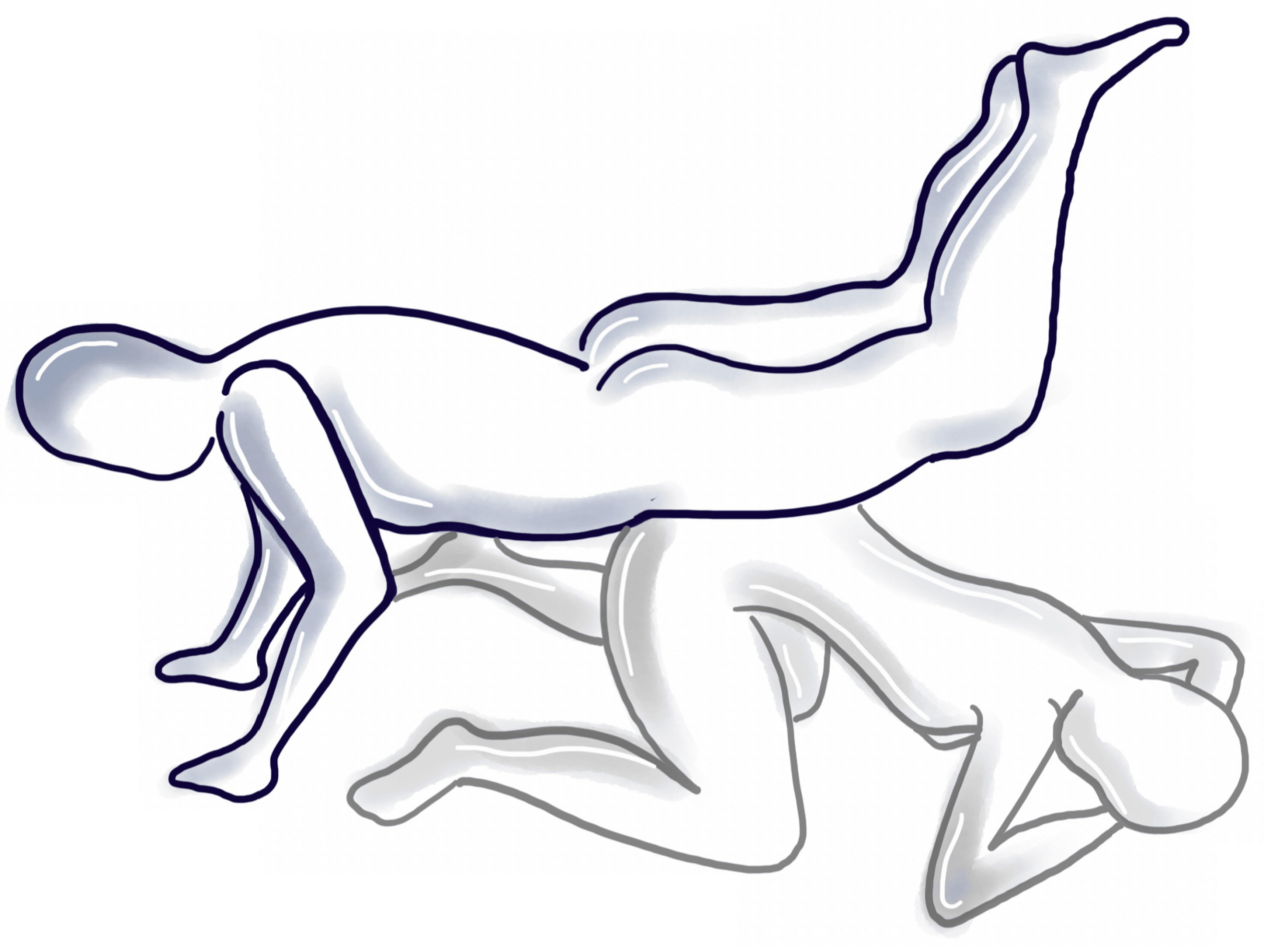
The "helicopter" position. This image is created by the author of this study.

In this sexual position, the receiving partner assumes a position on their hands and knees and then lowers themselves onto their elbows, creating a lower angle of the torso. The penetrating partner positions themselves beside the receiving partner, aligning their hips and facing the opposite direction toward the receiver’s feet. To achieve penetration, the penetrating partner lifts their legs over the receiving partner’s pelvis while maintaining their legs elevated in the air throughout the act (Figure 2) [6].

**Figure 3:**
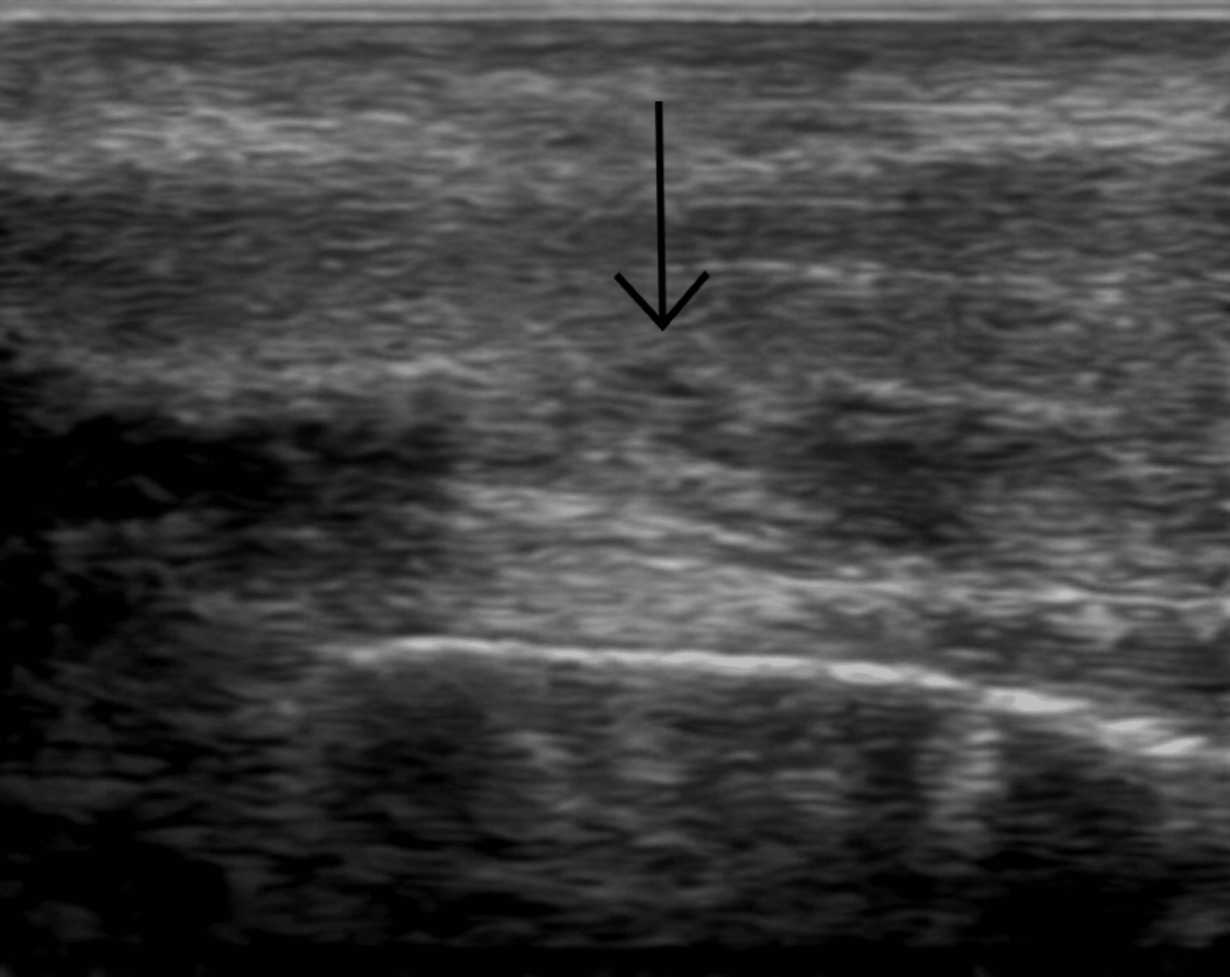
Ultrasound image of the penis revealing a 1 cm discontinuity in the tunica albuginea. The black arrow indicates the site of the tunica albuginea fracture.

Physical examination revealed significant edema and ecchymosis of the penile shaft, along with a palpable defect on its ventral aspect. Upon arrival at the emergency department, a penile duplex ultrasound was performed, which confirmed a fracture in the proximal third of the penis, associated with an 8.5 mL hematoma and an apparent branching component of the dorsal penile artery (Figure 3).

The patient received intravenous analgesics and empirical antibiotics as part of the initial medical management protocol. He was then taken to the operating room for exploration of the penis, evacuation of the hematoma, and primary closure of the tunica albuginea. Urine was diverted by the insertion of a suprapubic catheter. A direct longitudinal incision was made. The 1 cm defect was closed with 3-0 Monocryl, and the skin and subcutaneous tissue were closed with 2-0 Monocryl. The urethra had no damage. At the two-year follow-up, the patient was contacted and evaluated, with no evidence of erectile dysfunction or postoperative complications.

## Discussion

Penile fracture is a relatively rare urological emergency that requires prompt diagnosis and treatment to minimize long-term complications, such as erectile dysfunction and penile curvature. Full recovery of sexual function is the main goal of patients and urologists [7]. Proper performance of the penile duplex ultrasound, as well as a detailed physical examination and interrogation, is vital for diagnosis and prompt treatment [8]. Surgical management is the gold standard treatment, with good outcomes reported in the literature. However, the complexity of the penis poses formidable anatomical, functional, and cosmetic challenges when complications arise, which are often related to the surgical technique [9,10].

Pornography consumption has been studied for many years. Bőthe et al. in 2021 reported that the frequency of pornography use and the severity of problematic pornography use are associated with engaging in risky sexual behaviors. This is similar to our index case, in which a similar risky sexual position was undertaken, leading to a penile fracture. These behaviors may include experimenting with unusual or acrobatic sexual positions, prolonged intercourse sessions, and attempting to replicate acts that are not always anatomically safe. In individuals predisposed to impulsivity or sensation-seeking behaviors, these influences can significantly elevate the risk of traumatic sexual injuries, including penile fractures [5,11].

Moreover, the impact of easily accessible explicit material may contribute to the normalization of high-risk sexual practices among younger populations. The combination of limited sexual education and the lack of realistic depictions of safe sex in the media can foster unrealistic expectations and unsafe experimentation. Clinicians should consider addressing these behavioral factors during patient counseling and follow-up. Preventive strategies may include educational campaigns about the anatomical limits of the erect penis and the dangers of certain positions [12].

This case presents a penile fracture associated with vigorous sexual activity enhanced by pornography use and high-risk positions that can be easily found on the Internet. Increased awareness and education regarding safe sexual practices may help reduce the incidence of such injuries [13].

In addition to acute care, there is also a role for incorporating psychosexual education into urological practice, particularly in recurrent cases or those associated with identifiable behavioral patterns. Future research should explore the intersection of media consumption, sexual behavior, and genital trauma to inform more comprehensive prevention and intervention strategies.

Building on these realizations, prevention initiatives ought to go beyond the hospital. Together with educators and media outlets, urologists and sexual health specialists can advance truthful, anatomy-based sexual education that challenges the exaggerated depictions frequently found in pornography. The risk of trauma could be decreased by including information on safe sexual mechanics and the possible repercussions of using excessive force or adopting extreme positions in digital media and public health materials. Furthermore, incorporating conversations about consent and sexual safety into more comprehensive sexual education programs would promote healthier, better-informed behaviors and aid in the prevention of comparable injuries.

## Conclusions

Penile fracture is a urological emergency that requires prompt diagnosis and treatment to minimize long-term complications. Emerging evidence suggests that problematic pornography use may contribute to the risk of such injuries by encouraging individuals to engage in high-risk sexual behaviors or attempt anatomically hazardous positions depicted in explicit content.
